# Evaluation of the joint procedure pathway: Are we coding our activity?

**DOI:** 10.1111/ipd.13022

**Published:** 2022-09-12

**Authors:** Claudia Heggie, Annabelle Carter, Rosemary Bryan, Richard Balmer

**Affiliations:** ^1^ Paediatric Dentistry, School of Dentistry University of Leeds Leeds UK; ^2^ Paediatric Dentistry, Leeds Dental Institute Leeds Teaching Hospitals NHS Trust Leeds UK

## INTRODUCTION

Children with dental caries may be pre‐cooperative for dental treatment and often require pharmacological management with general anaesthesia (GA). The Getting It Right First Time (GIRFT) report for hospital dentistry found 29 588 children aged 5–9 years old had a dental general anaesthetic in 2018–2019.^1^ Hospital admission for dental reasons in children under 5 years old alone cost the National Health Service (NHS) £7.8 million in 2015–2016.^2^

As a specialised tertiary care unit situated adjacent to the Leeds Children's Hospital, the Paediatric Dentistry team at the Leeds Dental Institute cares for children with complex medical backgrounds. Many of these children are awaiting other medical investigations and procedures that require a GA. Each GA carries a small but significant risk of morbidity and mortality, which may be increased for children with complex medical backgrounds.

The attendance of the dental team to a medical GA to combine procedures can reduce the number of GAs experienced by each child. Recently, joint procedures or ‘piggybacks’ have been recommended in the GIRFT report to reduce the incidence of multiple GAs.^1^ Medical teams at the Leeds Children's Hospital, and specialist paediatric dentists within Yorkshire and the Humber, can refer children to the Paediatric Dentistry team at the Leeds Dental Institute for a joint procedure. Alternatively, the Paediatric Dentistry team can initiate this process. In 2014, a local audit found the quality of referrals from medical teams to be inconsistent and often lacking important information. A referral proforma was implemented, but its effectiveness had not been evaluated.

Often, joint procedures occur at short notice, without a prerequisite outpatient appointment as would be arranged for a procedure on a dedicated dental list.^3^ Treatment plans are often formulated during examination with general anaesthesia, and it is recommended that a specialist or consultant in paediatric dentistry is involved in this treatment planning.^1^, ^4^ For joint procedures, problems arise in staffing with appropriate supervision at short notice, which may result in understaffing of other departmental clinics.

A completed outcome sheet is required for joint procedure activity to be coded. The departmental diary is also utilised to record joint procedures but with minimal detail. The GIRFT report highlights the need for accurate reporting and coding of activity to allow evaluation of activity in addition to appropriate remuneration.^1^ Within our service, at time of writing, the tariff for a paediatric day case general anaesthetic for the extraction of multiple teeth was £835. For joint procedures, the procedure with the highest tariff, usually the paediatric surgical procedure, predominates. Accurate coding of this service, however, is important to demonstrate departmental activity for staffing, public health and commissioning purposes.

### Initial retrospective evaluation

Retrospective analysis of the departmental diary and outcome sheets for 2018–2019 found only nine completed outcome sheets compared with 27 recorded in the diary, with only three procedures documented in both sources. This discrepancy is important, as from the outcome sheet items of treatment are coded. This analysis therefore found 18 procedures that were not coded or remunerated in this initial period. Prospective evaluation was therefore planned to provide a more accurate representation of service utilisation that would not be reliant on previous note documentation.

## AIM

The aim of this study was to evaluate the scale and impact of joint procedures on our service in terms of staffing, resources and coding.

## OBJECTIVES

The following objectives were proposed:

- to prospectively determine the quality of joint general anaesthetic referrals;
- to prospectively evaluate the utilisation and provision of this service; and
- to prospectively evaluate the completion of outcome sheets and, by proxy, coding.

## METHODS

The project was registered locally prior to data collection. Prospective data collection occurred for the six‐month period from April to September 2021.

A data capture form was developed in accordance with previous locally agreed gold standard criteria for referral with additional outcomes to evaluate service utilisation (Table 1). Referrals were evaluated and awarded a percentage score for completeness. Data were inputted directly into Microsoft Excel 2021 (Version 16.56 Build 21121100) and descriptive statistics undertaken.

**TABLE 1:** Criteria for referral completeness and data captured for service evaluation

Referral criteria	Referrer details
	Patient identifiers (NHS number and date of birth)
	Relevant medical history
	Planned medical procedure
	Medical specialty and named consultant
	Planned procedure date
Service utilisation data	Date of referral, procedure and written dental consent
	Number and grade of staff attending
	Dental procedures completed
	Time taken for: preparation, waiting from team brief to procedure and dental procedural time
	Completion of outcome sheet

## STANDARDS

The following standards were considered:

- A standard of 100% was set for completeness of referrals and outcome sheets.
- No standard could be set for the evaluation of other outcomes.

## RESULTS

Over the six‐month period, 17 joint procedures occurred. The Paediatric Dentistry team was unable to staff two further joint procedures, and these cases were not included in further analyses. The mean age of patients was 7.6 years (range 4–15 years). The majority (94%, *n* = 16), of children had a degree of medical compromise. Five joint procedures had been initiated by the Paediatric Dentistry team. For the remaining 12 cases, a referral e‐mail was received: nine from medical teams and three from regional community dental services (CDS). No referrals utilised the proforma. Mean completeness of the referrals was 59% (range 50–70%), therefore not meeting the standard set.

Data regarding the number of working days between referral and joint procedure were non‐parametric, with a median value of 5 days (IQR 1.5–17.5). Most children (82%, *n* = 14) were dentally assessed prior to joint procedure; seven were assessed at the dental hospital and seven in CDS. Two children who were not assessed had an opportunistic examination under anaesthesia to enable future treatment planning. Medical specialties utilising this pathway were diverse, with joint procedures occurring with seven different medical specialties. Most parents (76.5%, *n* = 13) completed written consent for dental procedures on the same day as their child's GA.

The modal number of dentists to attend a joint procedure was two, a consultant attended 82.4% (*n* = 14) of cases. The modal number of dental nurses was also two. Most children, 82.4% (*n* = 14), received dental care during their joint procedure. Of these children, 28.6% (*n* = 4) received dental extractions only, and 71.4% (*n* = 10) received comprehensive dental care. Data for reported preparation time required for joint procedure were non‐parametric; the median preparation time was 35 min (IQR 30–53 min). Waiting time to perform treatment and time to complete dental treatment were normally distributed; mean waiting time was 146 min (*SD* = 70), and mean procedural time for dental treatment was 35 min (*SD* = 15.5 min). The mean total time was 227 min (*SD* = 103). A large variation in reported time was observed and is reflected in descriptive statistics for all time‐related variables. Of the 17 procedures completed in this period, outcome sheets and coding of activity were completed for 53% (*n* = 9). The activity was therefore only coded for just over half of all procedures.

## DISCUSSION

It was disappointing that no referrals had utilised the proforma. Ultimately, this resulted in inconsistency and poor completeness of referrals. This highlighted the importance of re‐auditing and closing the audit cycle to evaluate the previous proforma intervention, and this has been built this into our action plan. A surprising finding from this evaluation was the number of children referred by regional CDS. We have therefore incorporated these services into our dissemination plan.

On average, the paediatric dentistry team received less than a week's notice prior to planned joint procedures. This presents a significant challenge to the team in arranging staffing at short notice without impacting other departmental activities, particularly if these children require a dental assessment prior to their planned procedure. Additionally, this is exacerbated by the wide variation in time taken to deliver these joint procedures, making planning and staffing even more unpredictable and challenging. Ultimately, the paediatric dentistry team could not attend two joint procedures in the observed period, which represents avoidable repeat GAs for these children. A clinical member of staff has since been allocated to staffing these procedures, to enable prioritisation of joint procedure activity in line with the GIRFT report.^1^

This evaluation found joint procedures occupied on average nearly 4 hours of time from multiple staff members, with only half of procedures being coded. This is a substantial proportion of clinical time that is not coded, reported and potentially remunerated. When the paediatric dentistry team attend medical lists in different theatres, it may be that they omit to complete administrative processes associated with dental theatres. The impact on delivering a service that is not coded, however, is clear and may place financial implications on our ability to provide this vital service and on the wider NHS. Finally, poor documentation of these procedures precludes retrospective analysis for future service provision planning, research and quality improvement, which may limit service development.

## ACTION PLAN

The following action plan were proposed:

- The referral proforma has been updated and re‐circulated to medical teams and regional CDS.
- Allocated clinician to facilitate joint procedure staffing.
- Local dissemination of results with reminders to complete outcome sheets.
- Repeat evaluation is planned from September 2022 to March 2023.

## CONFLICT OF INTEREST

The authors declare no conflict of interest.
